# Dr. Bleecker Lansing Hovey

**Published:** 1906-06

**Authors:** 


### OBITUARY.

---

Dr. Bleecker Lansing Hovey, of Rochester, a graduate of Geneva Medical College, 1843, died at his home May 5, 1906, after a prolonged illness, aged 89 years. Dr. Hovey was surgeon of the 136th regiment New York Volunteer Infantry during the Civil War and served by detail as brigade and division Chief Surgeon in the Eleventh Army Corps. He was a member of several local and State Medical Societies and was for thirty years a Curator of the Medical Department of the University of Buffalo.

